# Improved delineation of the cystic artery using super-resolution deep learning reconstruction in contrast-enhanced abdominal computed tomography

**DOI:** 10.1007/s12194-026-01066-6

**Published:** 2026-05-18

**Authors:** Akira Katayama, Koichiro Yasaka, Yasunori Hamaguchi, Akiyoshi Hamada, Naomasa Okimoto, Osamu Abe

**Affiliations:** Department of Radiology, Graduate School of Medicine, The University of Tokyo, 7- 3-1 Hongo, Bunkyo-ku, Tokyo, 113-8655 Japan

**Keywords:** Super-resolution deep learning reconstruction, Cystic artery, Cystic duct, Deep learning, Proper hepatic artery

## Abstract

This study aimed to evaluate the image quality and delineation of the cystic artery and related abdominal vessels using Super-Resolution Deep Learning Reconstruction (SR-DLR) on contrast-enhanced computed tomography (CT), compared with standard Deep Learning Reconstruction (DLR). This retrospective study included 60 patients who underwent contrast-enhanced abdominal CT with an arterial phase between April and June 2025. Images were reconstructed using both SR-DLR and DLR algorithms. Quantitative evaluation included CT attenuation values, image noise, contrast-to-noise ratio (CNR), full width at half maximum (FWHM), and edge rise distance and edge rise slope (ERD/ERS) for the superior mesenteric artery (SMA) and proper hepatic artery (PHA). Three radiologists independently performed qualitative assessments of cystic artery, PHA, SMA, and cystic duct delineation, as well as image noise, sharpness, and artifacts. SR-DLR showed significantly lower image noise and higher CT attenuation values and CNR than DLR (*p* < 0.001). SR-DLR yielded higher ERS and lower ERD for the PHA and SMA compared to DLR (*p* ≤ 0.006). Qualitative analysis demonstrated that all three readers rated cystic artery delineation as significantly better with SR-DLR than with DLR (*p* ≤ 0.018). SR-DLR also achieved higher scores for image sharpness and diagnostic acceptability (*p* ≤ 0.001). SR-DLR significantly improves the delineation and image quality of the cystic artery and adjacent vascular anatomy compared with DLR. While no significant improvement in cystic artery detection was observed, the enhanced image quality may better support clinical confidence in preoperative planning for cholecystectomy and interventional radiology procedures.

## Introduction

The cystic artery is a small vessel, typically 1–2 mm in diameter, that most commonly arises from the right hepatic artery [1, 2]. Precise preoperative identification of the cystic artery is essential for cholecystectomy, particularly laparoscopic cholecystectomy [3]. The cystic artery often traverses Calot’s triangle (cystohepatic triangle), which is bounded by the cystic duct, the common hepatic duct, and the inferior surface of the liver. This region is characterized by complex anatomy and frequent anatomical variations [4, 5].

Unintentional cystic artery injury during surgery may result in hemorrhage that is difficult to control within the limited laparoscopic field [3]. Furthermore, bile duct injury represents a serious complication of laparoscopic cholecystectomy [6]. Although relatively uncommon, with an incidence of 0.3%–0.6%, bile duct injury can lead to bile leakage, infection, and jaundice, significantly worsening patient outcomes [5, 7]. Accordingly, detailed preoperative assessment of patient-specific vascular and biliary anatomy is recommended to reduce the risk of such complications [2–4].

Accurate preoperative identification of the cystic artery is also critical in Interventional Radiology, particularly for transarterial chemoembolization (TACE) in patients with hepatocellular carcinoma. Accidental embolization of the cystic artery during TACE may cause severe complications, including acute cholecystitis. Therefore, precise knowledge of the cystic artery origin and course before treatment is essential to prevent adverse events [8–10].

Historically, angiography was required to evaluate the cystic artery; however, this approach is invasive. Recent technical advances in multidetector computed tomography (CT) have enabled clear, noninvasive visualization of small peripheral vessels using contrast-enhanced CT [2, 3, 11]. Consequently, contrast-enhanced CT has become an important modality for preoperative anatomical assessment.

Recently, artificial intelligence (AI), including deep learning, has advanced rapidly in radiology. These technologies are applied not only to natural language processing [12] but also to Deep Learning Reconstruction (DLR) [13, 14]. Numerous studies have shown that DLR significantly reduces image noise and improves the signal-to-noise ratio (SNR) and contrast-to-noise ratio (CNR) compared with conventional reconstruction methods, such as Filtered Back Projection and Hybrid Iterative Reconstruction, thereby enhancing overall image quality and lesion conspicuity [15–18].

In abdominal imaging, DLR improves image quality not only in solid organs, such as the liver [14, 15], but also enhances the sharpness of major vessels, including the abdominal aorta and superior mesenteric artery (SMA) [19, 20]. Furthermore, DLR provides image quality sufficient for evaluating active bleeding in the abdominopelvic region [21].

More recently, Super-Resolution Deep Learning Reconstruction (SR-DLR), a novel technique that simultaneously reduces noise and enhances spatial resolution, has been introduced [22–25]. SR-DLR has been shown to improve delineation of medium-sized abdominal vessels, such as the proper hepatic artery (PHA), more effectively than DLR [26]. However, no studies have yet evaluated the usefulness of SR-DLR for visualizing the cystic artery, which is smaller, more peripheral, and characterized by substantial anatomical variation.

Therefore, the aim of this study was to investigate the effectiveness of SR-DLR in improving the delineation and subjective image quality of the cystic artery and related abdominal vessels compared with DLR on contrast-enhanced CT.

## Materials and methods

### Study population

This retrospective study was approved by the Institutional Review Board of our institution, and the requirement for informed consent was waived. A total of 117 patients who underwent contrast-enhanced upper abdominal CT between April 2025 and June 2025 were initially identified. The inclusion criterion was dynamic CT that included an arterial phase. Patients with a prior cholecystectomy (*n* = 56) and those with an indwelling gallbladder device (*n* = 1) were excluded. Consequently, 60 patients were included in the final study cohort.

### CT acquisition and reconstruction

All patients underwent abdominal CT examinations using a single scanner (Aquilion ONE INSIGHT Edition; Canon Medical Systems, Tokyo, Japan). Scan parameters were as follows: tube voltage, 120 kVp; tube current, automatic tube current modulation with a standard deviation of 13.0 for a 5-mm slice thickness; scan mode, helical; beam pitch, 0.813; gantry rotation time, 0.5 s; beam collimation, 40 mm; and detector collimation, 0.5 mm.

Contrast medium concentration and volume were adjusted according to body weight: 300 mgI/mL at body weight × 2 mL for patients < 50 kg; 350 mgI/mL at 100 mL for patients 50–60 kg; and 370 mgI/mL at 100 mL for patients > 60 kg. The contrast medium was injected through a peripheral vein within 30 s. Arterial-phase imaging was performed using a bolus-tracking technique, with a trigger threshold of 200 Hounsfield units (HU) in the descending aorta at the diaphragmatic level.

Arterial-phase axial and coronal images were reconstructed using two algorithms provided by Canon Medical Systems: Precise IQ Engine, an SR-DLR algorithm, and Advanced Intelligent Clear-IQ Engine, a DLR algorithm. The pixel matrix was 1024 × 1024 for SR-DLR and 512 × 512 for DLR. The field of view (350 mm, adjusted to patient physique) and slice thickness and interval (1 mm/1 mm) were identical for both reconstruction methods. A body kernel and strength level 2 were used for all reconstructions.

### Quantitative analysis

**Fig. 1:**
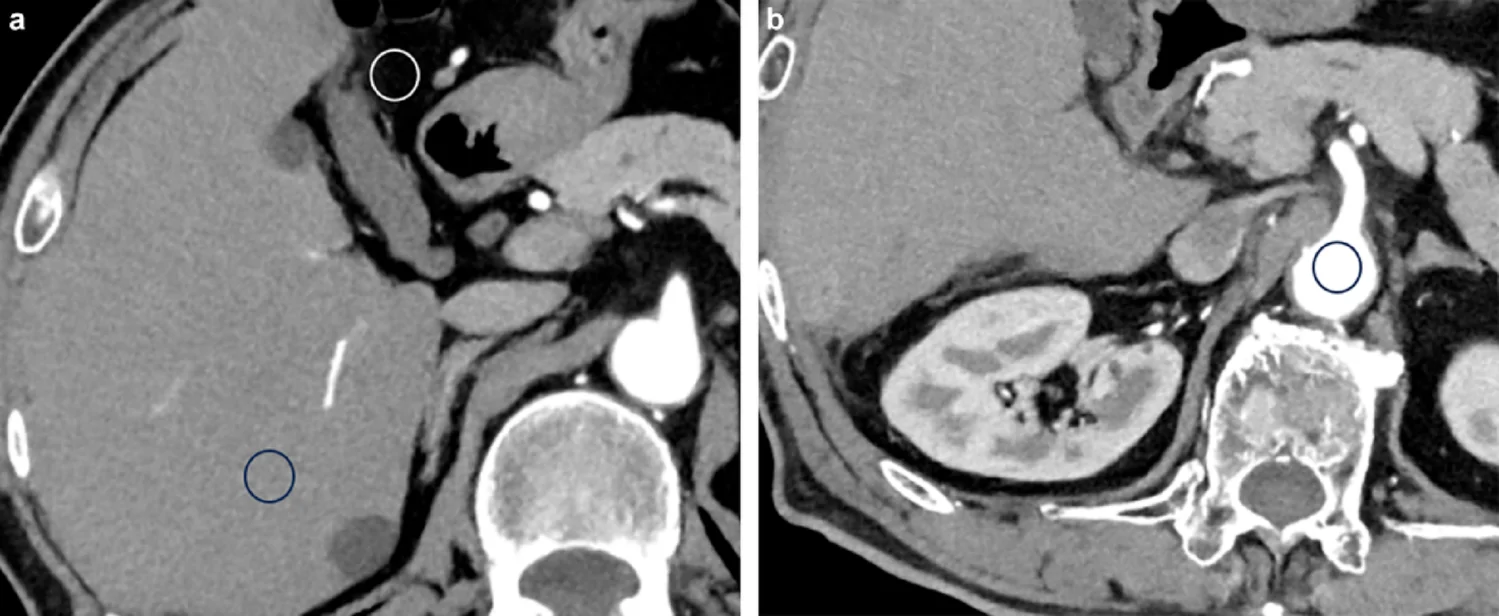
Circular region of interest was placed on the liver (black circle), visceral fat (white circle) (**a**), and abdominal aorta (black circle) (**b**) at the level of origin for the superior mesenteric artery

Under the supervision of a board-certified radiologist with 15 years of experience in abdominal radiology, a radiologist with 4 years of experience placed oval regions of interest (ROIs) of approximately 100 mm^2^ in the abdominal aorta at the level of the SMA origin, the liver parenchyma, and visceral fat (Fig. 1). ROIs were initially placed on SR-DLR images and then copied and pasted onto DLR images. Image noise was defined as the standard deviation (SD) of CT attenuation values in the liver parenchyma, abdominal aorta, and visceral fat.

The CNR between the liver parenchyma and visceral fat, and between the abdominal aorta and visceral fat, was calculated using the following formula:

$$*{\mathrm{CN}}{{\mathrm{R}}_{{\mathrm{ORGAN}}}}={{\left( {{\mathrm{M}}{{\mathrm{A}}_{{\mathrm{ORGAN}}}}-{\mathrm{M}}{{\mathrm{A}}_{{\mathrm{FAT}}}}} \right)} \mathord{\left/ {\vphantom {{\left( {{\mathrm{M}}{{\mathrm{A}}_{{\mathrm{ORGAN}}}}--{\mathrm{M}}{{\mathrm{A}}_{{\mathrm{FAT}}}}} \right)} {\sqrt {\left( {{{\left( {{\mathrm{S}}{{\mathrm{D}}_{{\mathrm{ORGAN}}}}^{{\mathrm{2}}}+{\mathrm{S}}{{\mathrm{D}}_{{\mathrm{FAT}}}}^{{\mathrm{2}}}} \right)} \mathord{\left/ {\vphantom {{\left( {{\mathrm{S}}{{\mathrm{D}}_{{\mathrm{ORGAN}}}}^{{\mathrm{2}}}+{\mathrm{S}}{{\mathrm{D}}_{{\mathrm{FAT}}}}^{{\mathrm{2}}}} \right)} 2}} \right. \kern-0pt} 2}} \right)} }}} \right. \kern-0pt} {\sqrt {\left( {{{\left( {{\mathrm{S}}{{\mathrm{D}}_{{\mathrm{ORGAN}}}}^{{\mathrm{2}}}+{\mathrm{S}}{{\mathrm{D}}_{{\mathrm{FAT}}}}^{{\mathrm{2}}}} \right)} \mathord{\left/ {\vphantom {{\left( {{\mathrm{S}}{{\mathrm{D}}_{{\mathrm{ORGAN}}}}^{{\mathrm{2}}}+{\mathrm{S}}{{\mathrm{D}}_{{\mathrm{FAT}}}}^{{\mathrm{2}}}} \right)} 2}} \right. \kern-0pt} 2}} \right)} }},$$

where MA_ORGAN_ and MA_FAT_ represent the mean CT attenuation values of the organ (liver parenchyma or abdominal aorta) and fat, respectively, and SD_ORGAN_ and SD_FAT_ represent the corresponding SD values. All measurements were performed using ImageJ software (https://imagej.net/ij/).

**Fig. 2:**
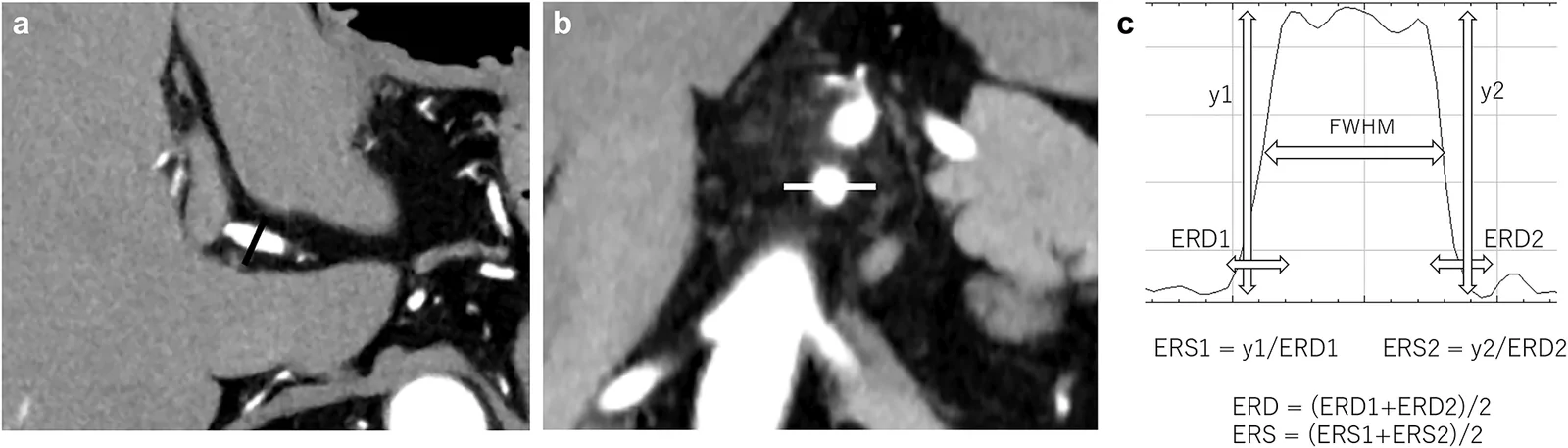
A linear region of interest was drawn perpendicularly across the PHA on the axial view (**a**) and SMA on the coronal view (**b**). Maximum computed tomography (CT) attenuation, full width at half maximum (FWHM), and slope at half maximum (average of the absolute values for the left and right slopes) were evaluated from the CT attenuation profile along the line regions of interest (**c**)

Furthermore, a linear ROI was placed across the center of the PHA on axial CT images (Fig. 2a) and across the center of the SMA origin on coronal images (Fig. 2b) to obtain CT attenuation profiles. From these line profiles, the maximum CT attenuation, the slope at the half-maximum value (calculated as the mean of the absolute left and right slopes), and the full width at half maximum (FWHM) were derived (Fig. 2c). ROI position and size were kept identical between reconstruction methods using a copy-and-paste function. Edge Rise Distance (ERD) was defined as the linear distance between the points where the signal intensity was 10% and 90% of the CT attenuation values difference between the maximum value (artery) and the minimum value (fat) on the profile. Edge Rise Slope (ERS) was defined as the maximum slope of the CT attenuation values profile at the edge, calculated by dividing the CT attenuation values difference by the ERD. FWHM was defined as the linear distance between the points where the CT attenuation values was 50% of the attenuation values difference between the maximum value (artery) and the minimum value (fat) on the profile. ERD and ERS values were averaged across both edges. Lower ERD and FWHM values, as well as higher ERS values, indicate better image sharpness [27].

### Cystic artery detection and qualitative image analyses

Before qualitative analysis, a radiologist with 15 years of experience randomized all image sets (SR-DLR and DLR). Three other radiologists (Readers 1, 2, and 3, with 7, 5, and 2 years of experience, respectively) performed qualitative image analysis using ImageJ software. They were blinded to patient background information and reconstruction algorithms. Images of patients excluded from the final analysis were used for training before the assessment to familiarize readers with the scoring system. They independently evaluated the images based on the following criteria:

First, for each patient, readers identified the number of cystic arteries. Examinations were divided into multiple sessions to ensure that SR-DLR and DLR images of the same patient did not appear in the same session. To prevent recall bias, sessions were separated by an interval of > 2 weeks. The cystic artery was defined as a nodular enhancing structure around the gallbladder merging with the common hepatic artery or its major branches, such as the right hepatic artery [3]. If a single cystic artery bifurcated into ≥ 2 branches, the termination was considered the bifurcation point. Both axial and coronal images were used for identification.

Based on the results of the reading experiment, the exact number of cystic arteries was determined via consensus between one board-certified radiologist with 15 years of experience and one radiology resident with 4 years of experience. Ten of the 60 eligible patients underwent abdominal angiography at our institution; angiography findings served as the reference standard for the number of cystic arteries in these patients. For patients without angiographic information (*n* = 50), the number of arteries was determined by consensus using all reconstruction images generated in this study (SR-DLR and DLR) and images generated for clinical use, serving as the gold standard.

The cystic artery identified using the reference standard was delineated using combined axial and coronal images. Scoring used a 4-point scale (4 = visible throughout the entire length (from origin to termination), 3 = more than two-thirds of the length is visible, but not the entire length, 2 = more than one-third but less than two-thirds of the length is visible, 1 = less than one-third of the length is visible).

The PHA and SMA, as well as the cystic duct, were delineated using axial and coronal images. For patients with a replaced right hepatic artery, the delineation of this artery was evaluated instead of the PHA. Delineation of the PHA and SMA was evaluated on a 4-point scale (4 = clear, 3 = slightly unclear areas present, 2 = unclear areas are prominent, 1 = poor delineation overall).

The cystic duct was delineated on a 4-point scale (4 = clear delineation, 3 = slightly unclear delineation, 2 = unclear delineation,1 = not delineated).

Image noise was evaluated on axial images, with a particular focus on areas with theoretically uniform CT values, such as inside blood vessels. Image noise was scored using a 4-point scale (4 = no or minimal noise, 3 = mild noise observed, 2 = noise is somewhat prominent, 1 = noise that could affect diagnosis is observed).

Sharpness was evaluated on axial images, focusing on the clarity of the margins of various structures. Sharpness was scored using a 4-point scale (4 = margins of structures are clear, 3 = margins of structures are slightly blurred in some areas, 2 = blurred margins are prominent, 1 = margins of structures are blurred overall).

Artifacts were evaluated using axial images. Scoring used a 4-point scale (4 = no or minimal artifacts, 3 = mild artifacts observed, 2 = artifacts are prominent but do not affect diagnosis, 1 = artifacts that could affect diagnosis are present).

Overall image quality was evaluated as diagnostic acceptability, determining whether the image quality was sufficient for the identification of the course of the cystic artery using combined axial and coronal images. Scoring used a 5-point scale (5 = image quality is sufficient for diagnosis, 4 = image quality is slightly better than standard, 3 = standard image quality/image quality is acceptable for diagnosis, 2 = image quality is slightly worse than standard, 1 = image quality is interfering with diagnosis).

All image interpretation experiments were conducted on a general PC using a single 27-inch color monitor (DELL U2718Q; 3840 × 2160, 60 Hz, 350 cd/m²). Although DICOM GSDF calibration was not formally performed, brightness and contrast were consistently set to 30% and 50%, respectively. The initial window width and level were standardized, but readers were allowed to manually adjust the window/level and magnification for optimal vessel tracing. Experiments were performed on the same terminal, with consistent ambient room lighting and viewing distance maintained throughout.

### Statistical analysis

Quantitative image comparisons between DLR and SR-DLR were performed using paired *t*-tests. Comparisons of the number of detected cystic arteries and subjective evaluations were conducted using the Wilcoxon signed-rank test. Accuracy rates were assessed using McNemar’s test. Inter-rater reliability among the three readers was evaluated using the intraclass correlation coefficient (ICC, model 2,1). To ensure the robustness of the scoring system, these coefficients were calculated using the combined dataset of all reconstruction methods. ICC values were interpreted as follows: ≤0.20, slight; 0.21–0.40, fair; 0.41–0.60, moderate; 0.61–0.80, substantial; and 0.81–1.00, almost perfect agreement. No adjustments for multiple comparisons were made because each qualitative image quality metric evaluates a distinct clinical characteristic. All statistical analyses were conducted using R (version 4.2.2; R Foundation for Statistical Computing, Vienna, Austria). *P*-values < 0.05 denote statistical significance.

## Results

The baseline characteristics are summarized in Table 1.

**Table 1:** Patient demographics

Patient	*n* = 60
Age, mean ± SD	69.5 ± 11.5
Sex (male/FEMALE)	50 / 10
Height (cm), mean ± SD	164.1 ± 7.9
Body weight (kg), mean ± SD	65.4 ± 15.0
Contrast volume (mL), mean ± SD	94.2 ± 5.7
Total iodine dose (mgI), mean ± SD	33,526.0 ± 3,172.6
Indications for CT	
Follow-up after treatment for HCC	32
Investigation of hepatic masses	12
Follow-up for metastatic tumors	8
Preoperative assessment for HCC	4
Investigation of liver cirrhosis	4
Abdominal Angiography, n	10
Indications for Angiography, n	
TACE for HCC	7
Hemostasis for GI bleeding	2
Coil embolization for visceral aneurysm	1
Interval from CT (days), median (range)	1350 (5832 before to 6 after)

*HCC* hepatocellular carcinoma, *SD* standard deviation, *TACE* transarterial chemoembolization, *GI* gastrointestinal

### Quantitative image quality analysis

Table 2 illustrates the quantitative evaluation results. Both image noise and CNR were significantly better with SR-DLR than with DLR (all *p* < 0.001). SR-DLR yielded higher ERS and lower ERD for the PHA and SMA compared to DLR (*p* ≤ 0.006). SR-DLR showed smaller FWHM than DLR for the SMA (*p* = 0.002), whereas no significant difference was observed between the two approaches for the PHA (*p* = 0.247). The CT attenuation values for the aorta, liver, and fat with the SR-DLR were significantly higher than those for the DLR (*p* < 0.001).

**Table 2:** Results of quantitative image analyses

	SR-DLR	DLR	Comparison
Linear profile			
Axial PHA			
ERD (mm)	2.3 ± 1.6	3.0 ± 1.5	0.006*
ERS (HU/mm)	163.0 ± 64.3	128.9 ± 57.9	< 0.001*
FWHM (mm)	4.6 ± 1.7	4.8 ± 2.0	0.247
Coronal SMA			
ERD (mm)	1.9 ± 1.2	2.4 ± 1.5	0.002*
ERS (HU/mm)	158.6 ± 55.2	134.3 ± 54.5	< 0.001*
FWHM (mm)	7.7 ± 1.9	7.8 ± 2.0	0.002*
Circular ROI			
CT attenuation value			
Liver (HU)	62.6 ± 10.5	59.9 ± 10.3	< 0.001*
Aorta (HU)	289.9 ± 44.7	284.9 ± 44.1	< 0.001*
Fat (HU)	-89.6 ± 17.5	-90.8 ± 17.3	< 0.001*
Noise			
Liver (HU)	8.5 ± 0.5	10.5 ± 1.0	< 0.001*
Aorta (HU)	8.9 ± 0.9	11.3 ± 1.4	< 0.001*
Fat (HU)	10.1 ± 1.3	11.9 ± 1.8	< 0.001*
CNR			
Liver	16.4 ± 2.6	13.5 ± 2.2	< 0.001*
Aorta	40.0 ± 6.4	32.5 ± 5.6	< 0.001*

*CNR* contrast-to-noise ratio, *DLR* deep learning reconstruction, *ERD* edge rise distance, *ERS* edge rise slope, *FWHM* full width at half maximum, *HU* Hounsfield unit, *SR-DLR* super-resolution deep learning reconstruction

*Statistically significant difference

### Cystic artery detection

**Fig. 3:**
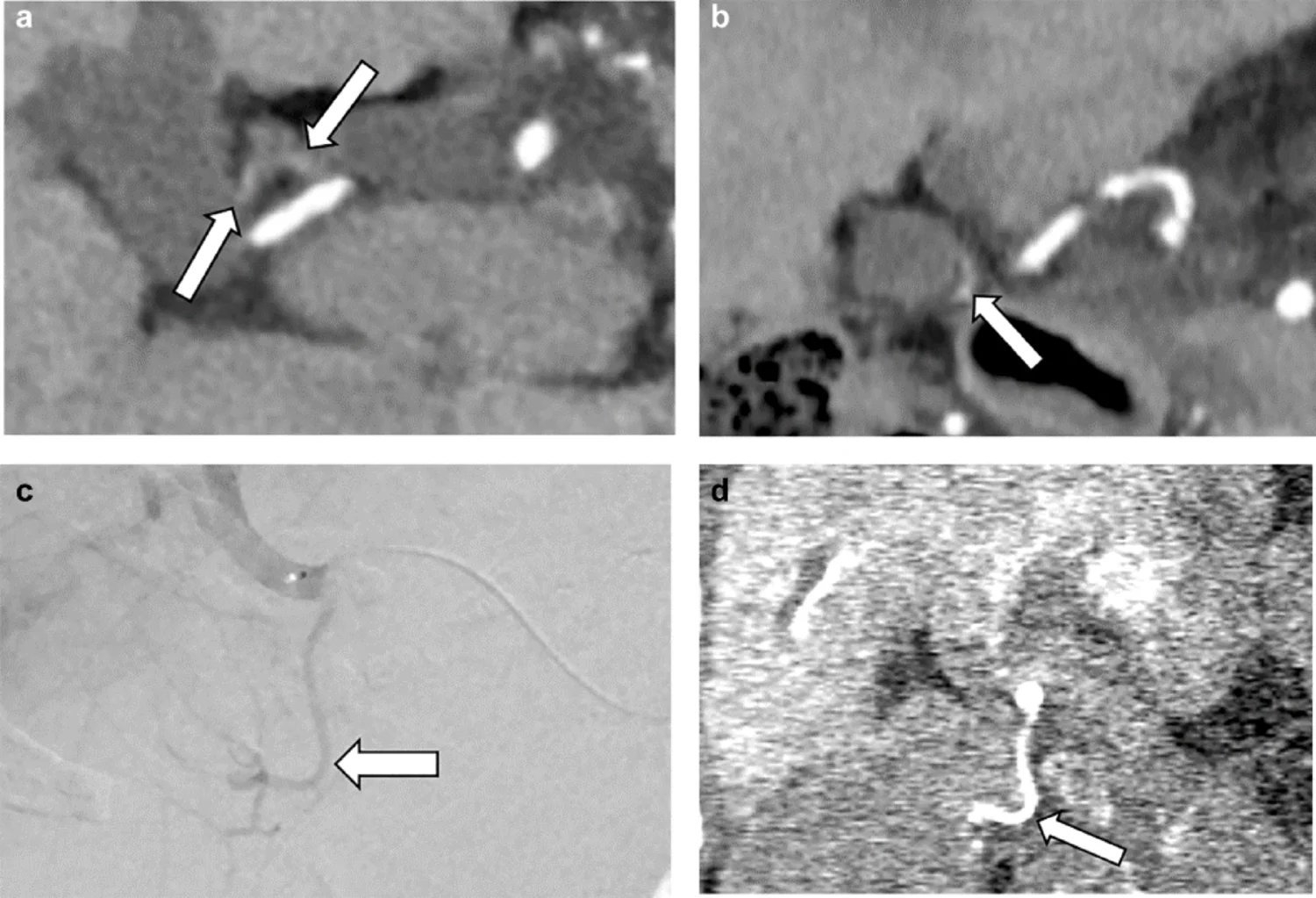
An 83-year-old woman undergoing treatment for hepatocellular carcinoma underwent abdominal angiography 6 days after contrast-enhanced CT. The arrows indicate the cystic artery. A single cystic artery was identified on the arterial-phase contrast-enhanced CT cross-sectional images (**a**) and coronal images (**b**) reconstructed with SR-DLR. A single cystic artery was also confirmed on right hepatic artery angiography (**c**) and intraoperative CT during hepatic arteriography (**d**)

Table 3 presents the number of cystic arteries detected by each reader. Seventy-eight cystic arteries were identified in the reference standard. The mean number of cystic arteries was 1.3 (no cystic artery detected in 3 cases, single artery in 36 cases, and two arteries in 21 cases). No cases presented with ≥ 3 cystic arteries. Although no significant differences in the number of detected cystic arteries or the accuracy rate were observed for any reader, SR-DLR tended to exhibit higher values than DLR (Fig. 3).

**Table 3:** Results of cystic artery detection analyses

	SR-DLR	DLR	Comparison
Reader 1			
Number of identified cystic arteries	67	62	0.096
Accuracy rate (%)	75.0	66.7	0.267
Reader 2			
Number of identified cystic arteries	73	73	1
Accuracy rate (%)	78.3	75.0	0.742
Reader 3			
Number of identified cystic arteries	66	58	0.106
Accuracy rate (%)	63.3	60.0	0.814

*DLR* deep learning reconstruction, *SR-DLR* super-resolution deep learning reconstruction

*Statistically significant difference

### Qualitative image quality assessment

**Fig. 4:**
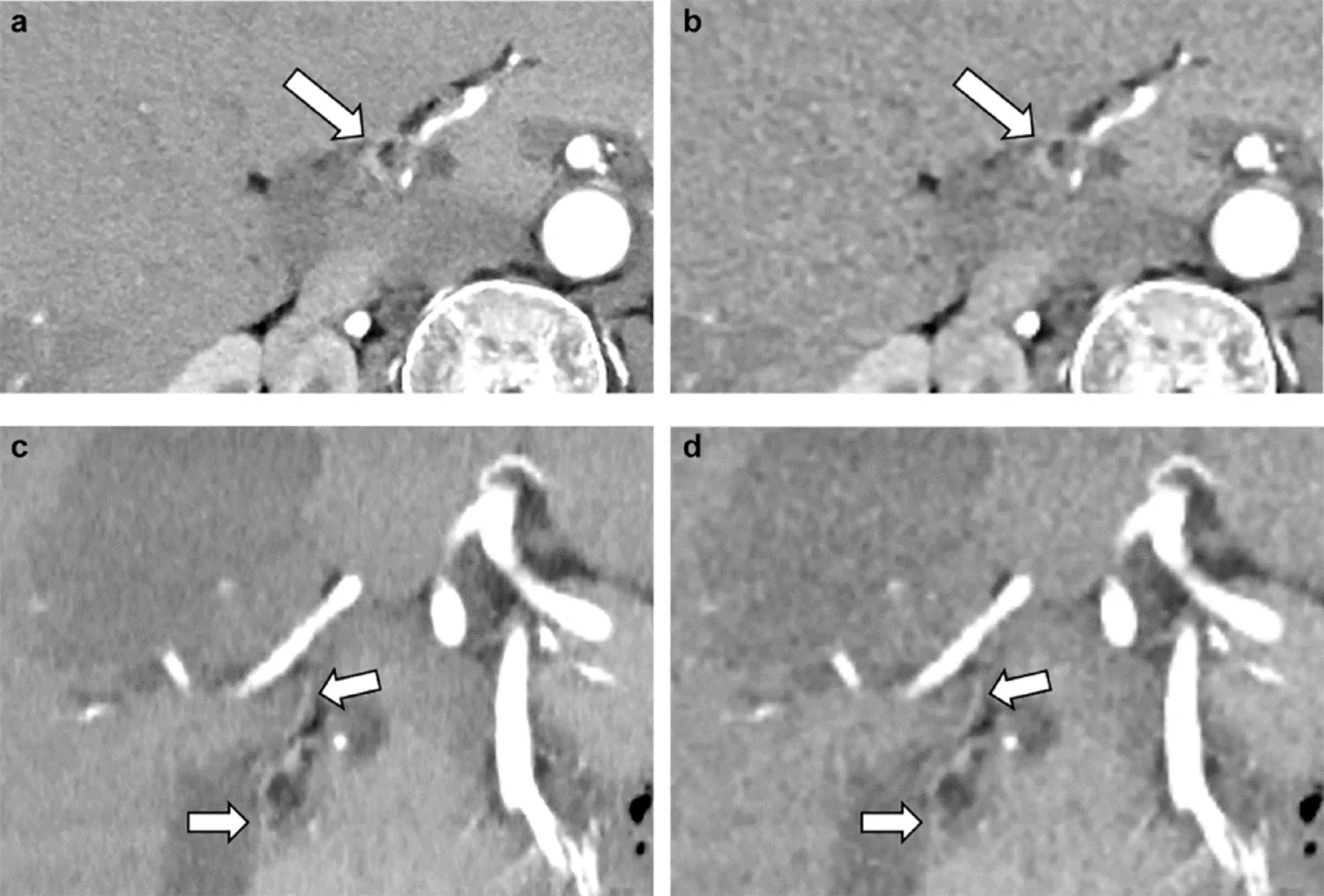
A 59-year-old woman underwent abdominal contrast-enhanced CT for follow-up of a giant hepatic hemangioma. The arrows indicate the cystic artery. The degree of depiction of the cystic artery (arrows) was rated by Readers 1, 2, and 3 as 4 (visible throughout the entire length [from origin to termination]), 4, and 3 (more than two-thirds of the length is visible, but not the entire length), respectively, in super-resolution deep learning reconstruction (**a**, **b**) and 3, 2 (more than one-third but less than two-thirds of the length is visible), and 2, respectively, in deep learning reconstruction (**c**, **d**)

**Fig. 5:**
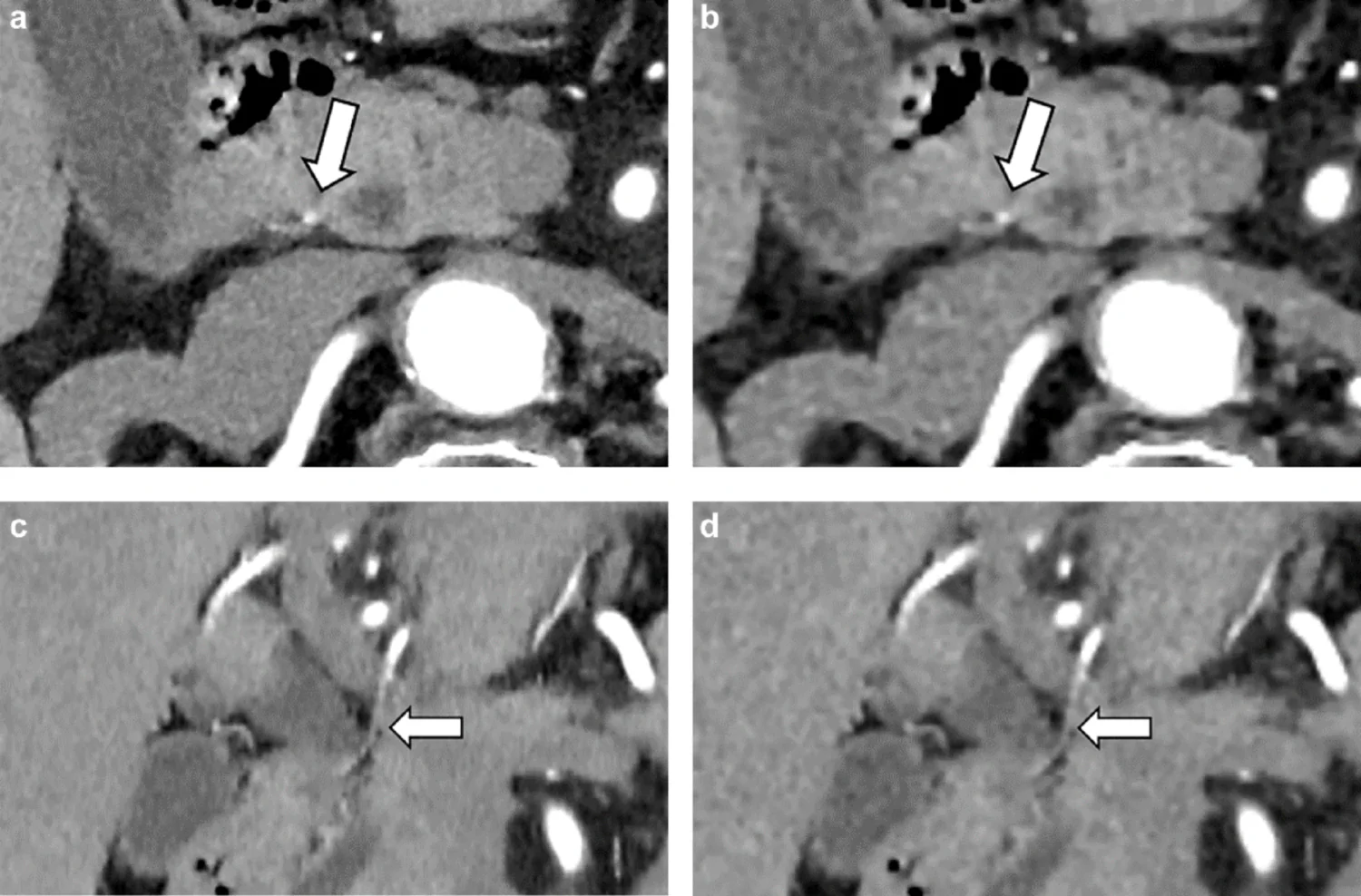
A 72-year-old man underwent abdominal contrast-enhanced CT for follow-up after radiofrequency ablation of hepatocellular carcinoma. The arrows indicate the cystic artery. The degree of depiction of the cystic artery (arrows) was rated by Readers 1, 2, and 3 as 4 (visible throughout the entire length [from origin to termination]), 4, and 3 (more than two-thirds of the length is visible, but not the entire length), respectively, in super-resolution deep learning reconstruction (**a**, **b**) and 3, 2 (more than one-third but less than two-thirds of the length is visible), and 2, respectively, in deep learning reconstruction (**c**, **d**)

Table 4 illustrates the results of the delineation evaluation. All three readers reported that SR-DLR exhibited better delineation ability for the cystic artery than DLR (Figs. 4 and 5) (*p* ≤ 0.018). Regarding the delineation of the cystic duct, Readers 1 and 2 evaluated SR-DLR as significantly better than DLR (*p* ≤ 0.002). For the delineation of the PHA and SMA, Reader 1 evaluated SR-DLR as significantly better than DLR (*p* < 0.001), whereas Readers 2 and 3 rated them as nearly equivalent. Regarding sharpness, noise, and diagnostic acceptability for vessel identification, all readers evaluated SR-DLR as significantly superior to DLR (*p* ≤ 0.001). Regarding artifacts, two readers regarded SR-DLR as superior to DLR (*p* < 0.001). The inter-rater reliability for the primary endpoints reached the level of moderate agreement. The highest ICC was observed in cystic duct depiction (ICC = 0.536), while depiction of the cystic artery (ICC = 0.441), image noise (ICC = 0.417), and diagnostic acceptability (ICC = 0.411) also showed moderate agreement. Sharpness showed fair agreement (ICC = 0.344). In the comparison of reconstruction methods, SR-DLR demonstrated clear superiority in key metrics across observers. However, some secondary items such as arterial depiction (PHA/SMA) and artifacts showed lower ICCs (0.070–0.150) and greater individual variability.

**Table 4:** Qualitative image analysis results

Reader	SR-DLR	DLR	Comparison	Inter-rater agreement (ICC)
Depiction of cystic artery				0.441
1	57/15/6/0	48/20/8/2	0.018*	
2	54/16/7/1	39/23/13/3	< 0.001*	
3	44/15/16/3	36/19/16/7	0.018*	
Depiction of cystic duct				0.536
1	24/35/1/0	15/37/8/0	0.001*	
2	46/9/4/1	33/17/8/2	0.002*	
3	36/16/7/1	29/22/8/1	0.107	
Depiction of PHA				0.126
1	51/9/0/0	22/38/0/0	< 0.001*	
2	53/5/2/0	47/12/1/0	0.144	
3	59/1/0/0	60/0/0/0	1	
Depiction of SMA				0.150
1	51/9/0/0	21/39/0/0	< 0.001*	
2	48/11/1/0	42/16/1/1	0.078	
3	59/1/0/0	60/0/0/0	1	
Sharpness				0.344
1	41/15/4/0	16/34/10/0	< 0.001*	
2	37/19/4/0	15/34/11/0	< 0.001*	
3	46/9/2/3	17/38/5/0	< 0.001*	
Noise				0.417
1	47/13/0/0	16/42/2/0	< 0.001*	
2	40/20/0/0	2/30/24/4	< 0.001*	
3	25/31/4/0	0/4/34/22	< 0.001*	
Artifact				0.070
1	47/13/0/0	21/39/0/0	< 0.001*	
2	18/40/2/0	6/38/16/0	< 0.001*	
3	52/6/2/0	53/3/3/1	0.588	
Diagnostic acceptability				0.411
1	20/35/5/0/0	5/32/23/0/0	< 0.001*	
2	30/25/5/0/0	7/16/22/13/2	< 0.001*	
3	15/16/21/8/0	9/9/26/16/0	0.001*	

The numbers of cystic arteries (for the depiction of cystic artery) and patients (for other metrics) with each score (5/4/3/2/1 or 4/3/2/1) are shown. DLR, deep learning reconstruction; ICC, Inter-rater agreement; PHA, proper hepatic artery; SMA, superior mesenteric artery; SR-DLR, super-resolution deep learning reconstruction.

*Statistically significant difference.

## Discussion

This study showed that thin-slice abdominal CT images reconstructed with SR-DLR provided better delineation of the cystic artery than those reconstructed with DLR for all readers. Two readers reported better delineation of the cystic duct with SR-DLR. Furthermore, abdominal CT reconstructed with SR-DLR demonstrated significantly lower subjective image noise and objectively higher CNR than abdominal CT reconstructed with DLR.

The cystic artery is a fine artery with a diameter of approximately 1–2 mm [1, 28], which is thinner than the PHA (approximately 4 mm). To the best of our knowledge, no previous study has investigated the delineation of fine abdominal arteries, such as the cystic artery, using SR-DLR. DLR improves the delineation of medium-to-small abdominal vessels [14, 15]. This study suggests that SR-DLR provides better delineation of the cystic artery than DLR. Although no statistically significant difference in the detection rate was observed, SR-DLR tended to have higher accuracy. Considering that the improved image quality (higher CNR and sharpness) contributes to better vessel conspicuity, SR-DLR may facilitate more confident identification of fine vascular structures. Regarding PHA evaluation, one reader reported that SR-DLR was significantly superior to DLR. This result is consistent with those of previous studies [26]. Furthermore, our study demonstrated that SR-DLR yielded significantly higher ERS and lower ERD for the linear profile of the PHA compared to DLR, indicating objectively improved delineation due to enhanced sharpness of vascular structural margins. The diameter of coronary arteries is approximately 3–4 mm, which is similar to that of the PHA [29]. In the thorax, SR-DLR has similarly been shown to enhance the delineation of coronary arteries compared with DLR [22, 30, 31]. Our results are consistent with these findings. In the objective evaluation, no significant difference in the FWHM of the PHA was observed between SR-DLR and DLR, which is consistent with previous reports measuring coronary arteries [22].

Regarding larger abdominal vessels, such as the SMA, subjective evaluation demonstrated that SR-DLR was significantly superior to DLR for one reader, whereas two readers rated them as comparable. In the objective evaluation of the SMA linear profile (ERS, ERD, and FWHM), SR-DLR exhibited enhanced sharpness of vascular margins compared with DLR. These results are consistent with previous reports [25]. We showed that SR-DLR improves delineation in the abdomen for vessels ranging from fine arteries, such as the cystic artery, to thick arteries, such as the SMA.

The visualization of the cystic duct using SR-DLR has not been previously reported. A study comparing DLR with conventional reconstruction methods using CT cholangiography reported that DLR improved the delineation of the cystic duct [32]. Within the same hepatobiliary-pancreatic region, SR-DLR has been demonstrated to provide better visualization of the pancreatic duct than DLR, and this finding was consistent with that report [25]. In the present study, the two more experienced readers (Readers 1 and 2) reported higher delineation capability with SR-DLR than with DLR. In contrast, the less experienced Reader 3 evaluated both reconstructions as comparable. This discrepancy likely arises because more experienced radiologists can more effectively perceive subtle improvements in image quality as an increase in clinical confidence.

The CT attenuation values for the aorta, liver, and fat with SR-DLR were consistently significantly higher than those with DLR. This trend was consistent with that observed in previous studies using SR-DLR [24, 26, 30]. Given that deep learning methods have a “black box” nature, the exact mechanism underlying this phenomenon remains unclear. DLR reduces noise and improves image quality, whereas SR-DLR additionally improves spatial resolution. Increasing resolution typically and inherently increases noise; however, subjective evaluation in this study revealed that noise was improved compared with DLR. Objective evaluation also supported this result. These results are consistent with those of previous studies [22, 25, 26], reporting that SR-DLR is superior to DLR in terms of spatial resolution and image noise. In this study, no difference in artifact evaluation was observed among raters, and two readers evaluated artifacts as significantly fewer in the subjective evaluation. Furthermore, one reader evaluated them as comparable. Regarding structural sharpness and diagnostic acceptability for tracing vessels, all readers evaluated SR-DLR as significantly superior to DLR. This is considered to reflect the effect of improving spatial resolution and reducing noise without increasing artifacts. The reliability of our subjective findings is reinforced by the ICC values for the primary endpoint. For the depiction of the cystic artery, the ICC was 0.441, indicating moderate agreement and confirming the reproducibility of our primary scoring system. In contrast, the ICC values for secondary endpoints, such as the depiction of the PHA and SMA, and artifacts, were notably lower (0.070–0.150), reflecting slight agreement among readers. This discrepancy may be attributed to a “ceiling effect”; since these larger vessels are typically well-visualized even with standard DLR, the subjective scores were clustered at the higher end of the scale, where minor individual variations in scoring thresholds disproportionately reduce the ICC. Furthermore, as observed in our results, the more experienced readers tended to perceive subtle enhancements in sharpness as clinical improvements, whereas the less experienced reader rated them as comparable. Despite this subjective variability in secondary metrics, the objective quantitative analysis—showing significantly higher ERS and lower ERD for both the PHA and SMA with SR-DLR—consistently demonstrated superior image sharpness. Therefore, while inter-rater agreement for larger vessels was limited by subjective thresholds, the collective evidence from both primary qualitative results and objective metrics supports the conclusion that SR-DLR provides a meaningful clinical advantage in the evaluation of fine and adjacent vascular structures.

This study has several limitations that should be acknowledged. First, because no surgical cases were included in this study, definitive confirmation of the reference standard was not obtained for all cases, except for those with a history of angiography. In cases without angiography, the reference standard was determined by consensus using all available CT images, including the SR-DLR reconstructions. We recognize that this approach introduces a degree of incorporation bias, which may inherently favor SR-DLR. Nevertheless, without invasive validation, we considered this consensus method to be the most practical way to estimate the true anatomy. Notably, cystic arteries used as the reference standard consisted of 21 cases with two arteries (35% of the total), which is comparable to the 28% reported in previous autopsy studies [28], indicating that the count was not inferior. Second, the cystic artery was too fine for objective evaluation using linear profiles. Because measurements such as the FWHM would be inaccurate because of the small sample size, subjective evaluation was considered clinically important. Third, the number of cases was limited. Further research is warranted. Fourth, the time interval between angiography and contrast-enhanced CT was substantial in several cases (median, 1,350 days). While the branching patterns and fundamental origins of the cystic artery are considered anatomically stable in adults unless surgical intervention or significant pathological changes (e.g., large tumor invasion) occur, this time lag could potentially affect the precise comparison of fine distal branches. However, to mitigate this, we also utilized a consensus of multiple CT reconstructions as a composite reference standard for cases with long intervals or without angiographic data. Fifth, although similar reconstruction algorithms are available from other vendors, the detailed mechanisms differ. Therefore, the results of this study cannot be directly applied to CT data acquired using scanners from other vendors.

In conclusion, SR-DLR significantly improved subjective and objective image quality for the delineation of the cystic artery and adjacent anatomy compared with DLR. Although cystic artery detection rates were comparable between the two algorithms, SR-DLR’s superior image quality and sharpness suggest it is a promising noninvasive tool for preoperative planning in cholecystectomy and interventional radiology.

## Data Availability

As the raw data for this study consist of clinical data containing personal information, they are not publicly available to protect individuals’ privacy but may be shared upon reasonable request.

## Declarations

### Declaration of Helsinki

All procedures performed in studies involving human participants were in accordance with the 1964 Helsinki Declaration and its later amendments.
